# Supplementary material for: Effects of acupuncture on earthquake survivors with major psychiatric disorders and related symptoms: A scoping review of clinical studies
Source: PLoS One. 2023 Jun 8;18(6):e0286671. doi: 10.1371/journal.pone.0286671 (PMC10249843; doi:10.1371/journal.pone.0286671)
Supplement: S1 Appendix — (DOCX) [file pone.0286671.s001.docx]

**S1 Table. Search terms used in each database.**

**Medline via PubMed**

|  | Searches | Results |
| --- | --- | --- |
| #1 | “earthquakes"[MeSH] OR earthquake[Title/abstract] OR “tsunamis"[MeSH] OR tsunami [Title/abstract] | 11068 |
| #2 | ("Acupuncture Therapy"[MeSH] OR "Acupuncture, Ear"[MeSH] OR "Acupuncture Points"[MeSH] OR "Acupuncture"[MeSH] OR "Electroacupuncture"[MeSH] OR "Meridians"[MeSH] OR acupuncture[Title/abstract] OR electroacupuncture[Title/abstract] OR electro-acupuncture[Title/abstract] OR acupoint*[Title/abstract]) | 36833 |
| #3 | #1 AND #2 | **15** |

**EMBASE via Elsevier**

|  | Searches | Results |
| --- | --- | --- |
| #1 | (‘earthquake’/exp OR earthquake OR ‘tsunami’/exp OR tsunami) | 14927 |
| #2 | ('acupuncture'/exp OR ‘acupuncture’ OR ‘acupuncture therapy’ OR ‘auricular acupuncture’/exp OR ‘auricular acupuncture’ OR ‘ear acupuncture’ OR ‘acupuncture point’/exp OR ‘acupuncture point’ OR ‘electroacupuncture’/exp OR ‘electroacupuncture’ OR ‘electro-acupuncture’ OR ‘body meridian’/exp OR ‘body meridian’ OR ‘acupoint’) | 65819 |
| #3 | #1 AND #2 | **25** |

**CENTRAL**

|  | Searches | Results |
| --- | --- | --- |
| #1 | MeSH descriptor: [earthquakes] explode all trees | 33 |
| #2 | (earthquake OR tsunami):ti,ab,kw | 176 |
| #3 | #1 OR #2 | 177 |
| #4 | MeSH descriptor: [Acupuncture] explode all trees | 166 |
| #5 | MeSH descriptor: [Acupuncture Therapy] explode all trees | 5377 |
| #6 | MeSH descriptor: [Acupuncture, Ear] explode all trees | 222 |
| #7 | MeSH descriptor: [Electroacupuncture] explode all trees | 899 |
| #8 | (Acupuncture OR Pharmacopuncture OR Ear acupuncture OR Pharmacoacupuncture OR Electroacupuncture OR Acupotomy OR Acupotomies OR Ear acupuncture OR Auricular acupuncture):ti,ab,kw | 18540 |
| #9 | #4 OR #5 OR #6 OR #7 OR #8 | 18679 |
| #10 | #3 AND #9 in Trials | 4 |

**Web of Science**

|  | Searches | Results |
| --- | --- | --- |
| #1 | (TS=(earthquake) OR TS=(tsunami)) | 183507 |
| #2 | (TS=(aupuncture therapy) OR TS=(acupuncture) OR TS=(acupuncture point) OR TS=(auricular acupuncture) OR TS=(ear acupuncture) OR TS=(electroacupuncture) OR TS=(electro-acupuncture) OR TS=(meridian) OR TS=(acupoint*)) | 45177 |
| #3 | #1 AND #2 | 104 |

**Scopus**

|  | Searches | Results |
| --- | --- | --- |
| #1 | (TITLE-ABS-KEY (earthquake) OR TITLE-ABS-KEY (tsunami) | 244574 |
| #2 | (TITLE-ABS-KEY (aupuncture therapy) OR TITLE-ABS-KEY (acupuncture) OR TITLE-ABS-KEY (acupuncture point) OR TITLE-ABS-KEY (auricular acupuncture) OR TITLE-ABS-KEY (ear acupuncture) OR TITLE-ABS-KEY (electroacupuncture) OR TITLE-ABS-KEY (electro-acupuncture) OR TITLE-ABS-KEY (meridian) OR TITLE-ABS-KEY (acupoint*)) | 69635 |
| #3 | #1 AND #2 | 98 |

**AMED via EBSCO**

|  | Searches | Results |
| --- | --- | --- |
| #1 | (earthquake[TX] OR tsunami[TX]) | 82 |
| #2 | (“Acupuncture Therapy”[SU] OR “Acupuncture, Ear”[SU] OR “Acupuncture Points”[SU] OR Acupuncture[SU] OR Electroacupuncture[SU] OR Meridians[SU] OR acupuncture[TX] OR electroacupuncture[TX] OR electro-acupuncture[TX] OR acupoint*[TX]) | 12294 |
| #3 | #1 AND #2 | 3 |

**CINAHL via EBSCO**

|  | Searches | Results |
| --- | --- | --- |
| #1 | (earthquake[TX] OR tsunami[TX]) | 9755 |
| #2 | (“Acupuncture Therapy”[MH] OR “Acupuncture, Ear”[MH] OR “Acupuncture Points”[MH] OR Acupuncture[MH] OR Electroacupuncture[MH] OR Meridians[MH] OR acupuncture[TX] OR electroacupuncture[TX] OR electro-acupuncture[TX] OR acupoint*[TX]) | 36644 |
| #3 | #1 AND #2 | 95 |

**PsycARTICLES via ProQuest**

|  | Searches | Results |
| --- | --- | --- |
| #1 | (earthquake OR tsunami) | 1966 |
| #2 | mesh(Acupuncture Therapy) OR mesh(Acupuncture, Ear) OR mesh(Acupuncture Points) OR mesh(Acupuncture) OR mesh(Electroacupuncture) OR mesh(Meridians) OR ‘acupuncture’ OR ‘electroacupuncture’ OR ‘electro-acupuncture’ OR acupoint* | 376 |
| #3 | #1 AND #2 | 13 |

**OASIS**

|  | Searches | Results |
| --- | --- | --- |
| #1 | (지진 OR 해일) AND 침 | **0** |

**KCI**

|  | Searches | Results |
| --- | --- | --- |
| #1 | (지진 OR 해일) AND 침 | **51** |

**CNKI**

|  | Searches | Results |
| --- | --- | --- |
| #1 | (SU=''地震'+'海啸') AND (SU='acupuncture'+'针'+'鍼') | **110** |

**Wanfang data**

|  | Searches | Results |
| --- | --- | --- |
| #1 | 主题:(‘'地震'+'海啸') * 主题:("acupuncture" + "针" + "鍼") | **2981** |

**VIP**

|  | Searches | Results |
| --- | --- | --- |
| #1 | (M=(地震 OR 海啸 (M=(acupuncture OR 针 OR 鍼)) | **28** |

**CiNii**

|  | Searches | Results |
| --- | --- | --- |
| #1 | (地震 OR 津波) AND (acupuncture OR 針 OR 鍼)) | **916** |
