# Supplementary material for: Effects of acupuncture on earthquake survivors with major psychiatric disorders and related symptoms: A scoping review of clinical studies
Source: PLoS One. 2023 Jun 8;18(6):e0286671. doi: 10.1371/journal.pone.0286671 (PMC10249843; doi:10.1371/journal.pone.0286671)
Supplement: S2 Appendix — (DOCX) [file pone.0286671.s002.docx]

**Supplemental Digital Content** 2**. Quality assessment results of the included studies**

1. RCTs – Cochrane’s risk of bias tool

| Study | 1. Random sequence generation | 2. Allocation concealment | 3. Blinding of participants and personnel | 4. Blinding of outcome assessment | 5. Incomplete outcome data | 6. Selective reporting | 7. Other sources of bias |
| --- | --- | --- | --- | --- | --- | --- | --- |
| Zhang 2010-a | Low risk | Low risk | High risk | Low risk | Low risk | Low risk | Low risk |
| Zhang 2010-b | Low risk | Unclear | High risk | Unclear | Low risk | Low risk | Low risk |
| Zhang 2010-c | Unclear | Unclear | High risk | Unclear | Low risk | Low risk | Low risk |
| Zhao 2014 | High risk | Unclear | High risk | Unclear | Low risk | Low risk | Low risk |

2. Before-after studies - Quality Assessment Tool for Before-After (Pre-Post) Studies With No Control Group (proposed by the National Heart, Lung, and Blood Institute)

| Study | *1. Was the study question or objective clearly stated?* | *2. Were eligibility/selection criteria for the study population prespecified and clearly described?* | *3. Were the participants in the study representative of those who would be eligible for the test/service/intervention in the general or clinical population of interest?* | *4. Were all eligible participants that met the prespecified entry criteria enrolled?* | *5. Was the sample size sufficiently large to provide confidence in the findings?* | *6. Was the test/service/intervention clearly described and delivered consistently across the study population?* | *7. Were the outcome measures prespecified, clearly defined, valid, reliable, and assessed consistently across all study participants?* | *8. Were the people assessing the outcomes blinded to the participants' exposures/interventions?* | *9. Was the loss to follow-up after baseline 20% or less? Were those lost to follow-up accounted for in the analysis?* | *10. Did the statistical methods examine changes in outcome measures from before to after the intervention? Were statistical tests done that provided p values for the pre-to-post changes?* | *11. Were outcome measures of interest taken multiple times before the intervention and multiple times after the intervention (i.e., did they use an interrupted time-series design)?* | *12. If the intervention was conducted at a group level (e.g., a whole hospital, a community, etc.) did the statistical analysis take into account the use of individual-level data to determine effects at the group level?* |
| --- | --- | --- | --- | --- | --- | --- | --- | --- | --- | --- | --- | --- |
| Wang 2009 | Yes | No | Yes | Cannot determine | Cannot determine | Yes | No | Cannot determine | Not applicable | Not applicable | Not applicable | Not applicable |
| Yuan 2009 | Yes | No | Yes | Cannot determine | Cannot determine | Yes | No | Cannot determine | Not applicable | Not applicable | Not applicable | Not applicable |
| Li 2012 | Yes | Yes | Yes | Cannot determine | Cannot determine | Yes | Yes | Yes | Not applicable | Yes | Yes | Not applicable |
| Moiraghi 2019 | Yes | No | Yes | Cannot determine | Cannot determine | No | No | Cannot determine | Not applicable | Yes | Yes | Not applicable |
| Kim 2020 | Yes | No | Yes | Cannot determine | Cannot determine | Yes | Yes | Cannot determine | No | Yes | Yes | Not applicable |
